# Knockdown of angiopoietin-like 2 induces clearance of vascular endothelial senescent cells by apoptosis, promotes endothelial repair and slows atherogenesis in mice

**DOI:** 10.18632/aging.102020

**Published:** 2019-06-11

**Authors:** Laurie Caland, Pauline Labbé, Maya Mamarbachi, Louis Villeneuve, Gerardo Ferbeyre, Pierre-Emmanuel Noly, Michel Carrier, Nathalie Thorin-Trescases, Éric Thorin

**Affiliations:** 1 Faculty of Medicine, Department of Pharmacology and Physiology, Université de Montréal, Montreal, Quebec, Canada; 2 Montreal Heart Institute, Université de Montréal, Montreal, Quebec, Canada; 3 Faculty of Medicine, Department of Biochemistry, Université de Montréal and CRCHUM, Montreal, Quebec, Canada; 4 Faculty of Medicine, Department of Surgery, Université de Montréal, Montreal, Quebec, Canada

**Keywords:** p21, Bax/Bcl2, CD34, PAI-1, human internal mammary artery

## Abstract

Elimination of senescent cells (SnC) is anti-atherogenic, but the specific contribution of senescent vascular endothelial cells (EC) is unknown. We inactivated angiopoietin like-2 (angptl2), a marker of SnEC and a pro-atherogenic cytokine in LDLr^-/-^, hApoB_100_^+/+^ atherosclerotic (ATX) mice. Three months after a single vascular delivery of a small hairpin (sh)Angptl2 in 3-month old ATX mice using an adeno-associated virus serotype 1 (AAV1), aortic atheroma plaque progression was slowed by 58% (p<0.0001). In the native aortic endothelium, *angptl2* expression was decreased by 80%, in association with a reduced expression of *p21*, a cyclin-dependent kinase inhibitor overexpressed in growth-arrested SnC. Endothelial activation was reduced (lower *Icam-1, Il-1β* and *Mcp-1* expression), decreasing monocyte *Cd68* expression in the endothelium. One week post-injection, the ratio *Bax/Bcl2* increased in the endothelium only, suggesting that *angptl2^+^/p21^+^* SnEC were eliminated by apoptosis. Four weeks post-injection, the endothelial progenitor marker *Cd34* increased, suggesting endothelial repair. In arteries of atherosclerotic patients, we observed a strong correlation between *p21* and *ANGPTL2* (r=0.727, p=0.0002) confirming the clinical significance of *angptl2*-associated senescence. Our data suggest that therapeutic down-regulation of vascular *angptl2* leads to the clearance of SnEC by apoptosis, stimulates endothelial repair and reduces atherosclerosis.

## Introduction

Senescent cells lose their proliferative potential in response to various stresses. They secrete a variety of pro-inflammatory mediators and proteases, gathered in the senescence-associated secretory phenotype (SASP) [1] that engages the immune system to eliminate senescent cells [2,3]. Senescent cells accumulate in aging organisms, chronic age-related diseases and benign tumors [4–7]; conversely, elimination of senescent cells contributes to improve health [8–11]. They also accumulate in tissues affected by atherosclerosis [12–14] and their elimination strikingly reduces atherogenicity in animal models [12,14]. Senescence is thus a link between molecular damage and the altered physiology of aging, and targeting SnC using senolytic drugs appears a promising strategy to reduce the burden of age-related chronic inflammatory diseases [15], including atherosclerosis [16,17]. Selective and safe senolytics, however, have yet to be discovered as most have emerged from the oncology therapeutic armamentarium [18]. Combination of the natural product quercetin with the tyrosine kinase inhibitor dasatinib preferentially killed senescent cells in culture and in mice, improving health span in naturally aged mice [19,20]. ABT263, a Bcl2 family inhibitor, was able to eliminate senescent cells after irradiation in mice and to rejuvenate bone marrow stem cells from both irradiated mice and naturally aged mice [21], to reverse pulmonary fibrosis in a mouse model [22], and to reduce senescence-associated Tau-dependent neuronal damage and cognitive decline [23].

Angiopoietin like-2 (angptl2) is a member of the SASP [24–28] and is detectable in most organs of adult mice [29]. Angptl2 is expressed by senescent vascular human endothelial cells (EC) [30], but not quiescent or proliferative EC [31] and is atherogenic when infused in young LDLr^-/-^;hApoB_100_^-/-^ atherosclerotic (ATX) mice [31] or in angptl2-endothelial transgenic ApoE^-/-^ mice [32]. We reported that plasma levels of angptl2 are elevated in patients with cardiovascular diseases (CVD) [31], were associated with endothelial dysfunction [33] and were predictive of major cardiac adverse events (MACE) and death [34]. Recently, we reported a strong relationship between arterial expression of *p21*, a cell cycle inhibitor overexpressed in senescent cells and maintaining growth arrest [35], and circulating levels of angptl2 in atherosclerotic patients [36]. Senescent EC are activated and promote aggregation of leukocytes [37], the initiating step of atherogenesis [38]. We therefore hypothesized that down-regulation of vascular *angptl2*, preferentially in the endothelium of severely dyslipidemic ATX mice would promote endothelial repair and slow atherogenesis. Here, we report that knockdown of vascular *angptl2* by a shRNA (shAngptl2), delivered to the vascular cells *via* a single injection of an AAV1 [39], slowed atheroma progression in ATX mice. Knockdown of angptl2 was associated with a rapid reduction in the expression of EC senescence-associated *p21* accompanied by the increase in *Bax/Bcl2* ratio as a marker of apoptosis; subsequently, this was associated with endothelial repair as evidenced by the incorporation of endothelial progenitor CD34^+^ cells. In addition to our pre-clinical results, we show that vascular *ANGPTL2* gene expression is correlated with *p21* expression and inflammatory cytokines in the internal mammary artery isolated from severely atherosclerotic patients undergoing a coronary artery bypass surgery. Altogether, our data suggest that targeting vascular *angptl2* could be senolytic, delaying the progression of atherosclerosis.

## RESULTS

### Endothelial expression of angptl2 and senescence gene markers parallels atherogenesis

**Figure 1:**
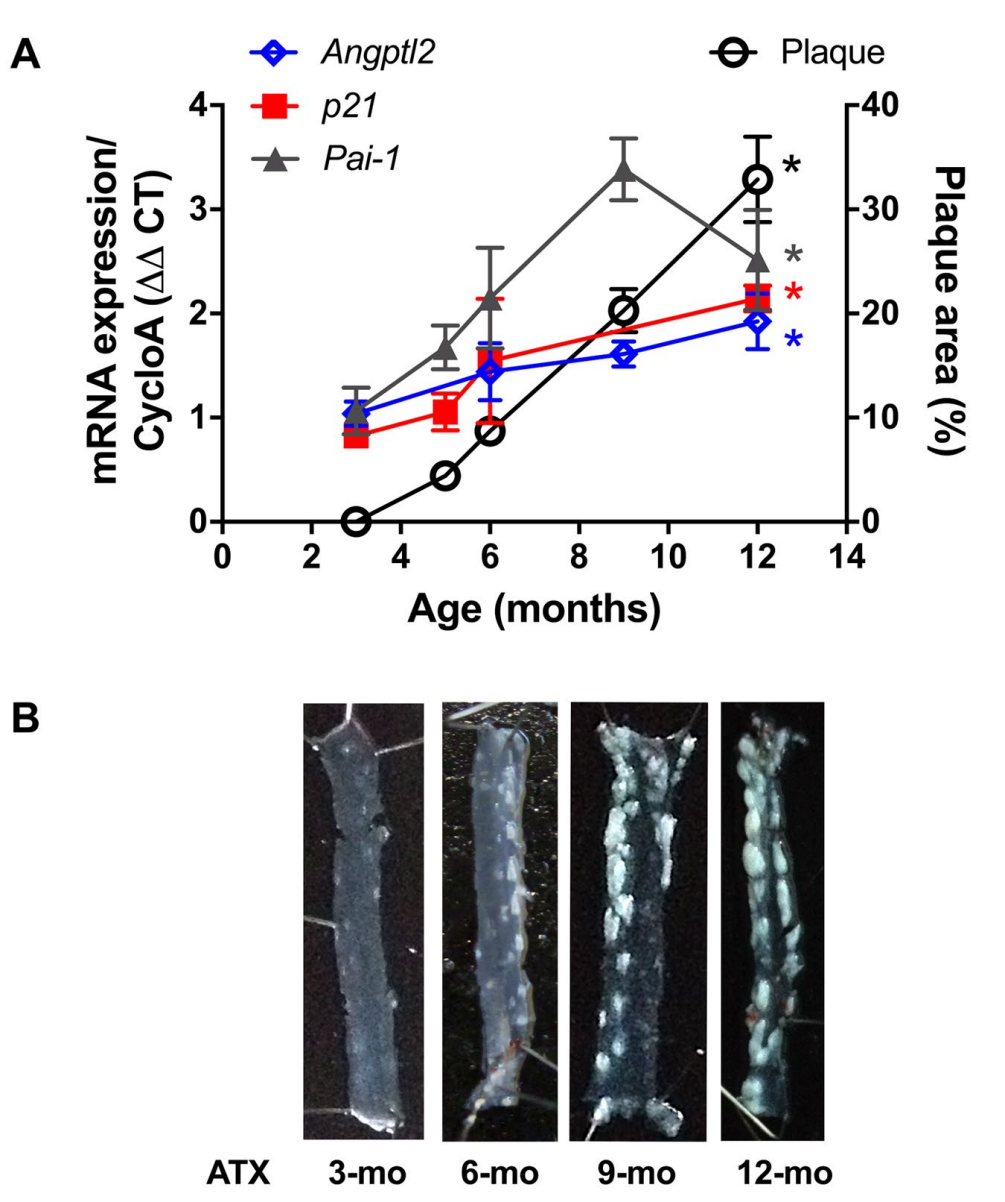
**Age-dependent increase of senescence-associated *p21, Pai-1* and *angptl2* expressions in the native endothelium parallels plaque growth in the aorta.** (**A**) mRNA expression of indicated genes was quantified in the native aortic endothelium of 3-mo (n=4), 5-mo (n=4), 6-mo (n=4) and 12-mo (n=4) control ATX mice. The average level of gene expression in 3-mo ATX mice was arbitrarily set at 1. Plaque area was quantified from longitudinally open thoracic aortas of 3-mo (n=7), 5-mo (n=5), 6-mo (n=7), 9-mo (n=12) and 12-mo (n=4) ATX mice. Data are expressed as mean±SEM. *: p<0.0001 *vs.* 3-mo ATX mice. (**B**) Representative pictures of age-related increase in atherosclerotic plaque in 3-, 6-, 9- and 12-mo ATX mice.

**Figure 2:**
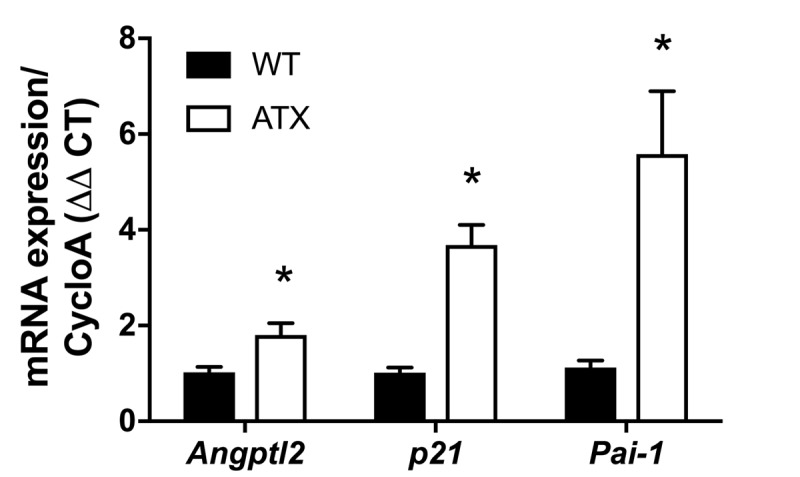
**Increased expression of senescence-associated *p21, Pai-1* and *angptl2* in the native aortic endothelium of 6-month old ATX compared to WT mice.** mRNA expression of indicated genes was quantified in the native aortic endothelium of 6-mo WT and ATX mice (n=3). The average level of gene expression in 6-mo WT mice was arbitrarily set at 1. Data are expressed as mean±SEM. *: p<0.05 *vs.* WT mice.

Firstly, and as expected, endothelial expression of *angptl2, Pai-1* and *p21* parallels the growing atheroma plaque in untreated LDLr^-/-^;hApoB_100_^+/+^ atherosclerotic (ATX) mice up to 12-month old (-mo) (Figure 1); *p21* is a cyclin-dependent kinase inhibitor overexpressed in growth-arrested senescent cells, and *Pai-1* is a recognized SASP member and inducer of senescence [15]. When compared to age-matched wild-type mice, *angptl2, Pai-1* and *p21* are over-expressed in the native endothelium of 6-mo ATX mice (Figure 2).

### Vascular *angptl2* knockdown decreases atherosclerotic plaque size

**Figure 3:**
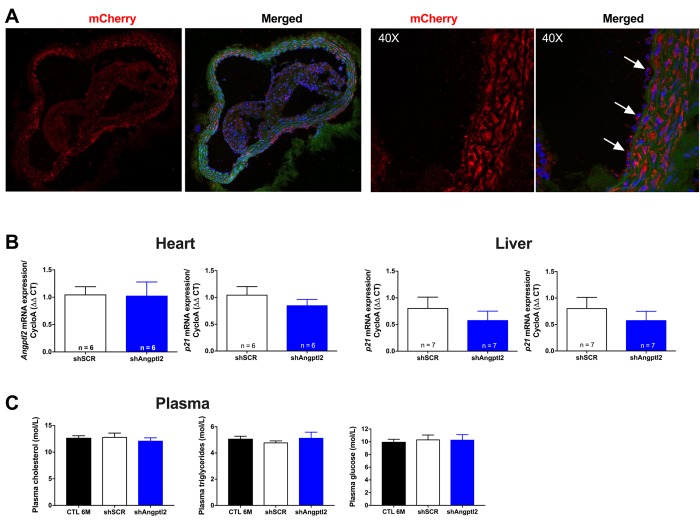
**Distribution of the AAV1-mCherry in the aortic wall and specificity of the AAV1-shAngptl2.** (**A**) Immunofluorescence of AAV1-mCherry in frozen aortic sections of ATX mice at 6 months of age, 3 months post-infection: mCherry signal distributed throughout the vascular wall is shown in red and basal lamina in green; nuclei are shown in blue. At a higher magnification (40X), arrows show mCherry signal in the endothelium. A negative control (absence of primary antibody against mCherry) was performed (data not shown). (**B**) Neither cardiac nor liver *Angptl2* and *p21* mRNA expressions were affected by the AAV1-shAngptl2 in ATX mice, 3 months post-infection. Average gene expression level in shSCR mice was arbitrarily set at 1. Data are mean±SEM of *n* ATX mice. **C**) Cholesterol, triglycerides and glucose levels of ATX mice were not altered by the AAV1-shAngptl2, 3 months post-infection. Data are mean±SEM of n=7 ATX mice.

To investigate the anti-atherogenic effects of *angptl2* knockdown, we delivered once a shAngptl2 (Table S1) using an adeno-associated virus serotype 1 (AAV1) as a vector (i.v. bolus injection) with preferred vascular tropism [39] in 3-mo ATX mice. Each mouse was sacrificed at 6-mo. The vascular delivery of the shRNA was confirmed by mCherry staining of the aortic wall, showing red fluorescence in the endothelial cells and throughout the vascular wall, but with no diffusion to the adventitia or in the plaque (Figure 3A). In addition, the AAV1-shAngptl2 infection neither reduced *angptl2* expression in the mouse heart and liver (Figure 3B), nor affected lipid and glucose blood levels (Figure 3C).

**Figure 4:**
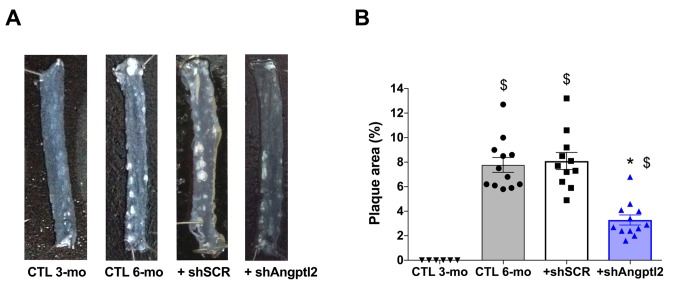
**Down-regulation of vascular *angptl2* is anti-atherogenic.** (**A**) Representative images of longitudinally open thoracic aortas showing atherosclerotic plaque, and (**B**) Quantification of thoracic aorta atheroma plaque area in 3 mo control (CTL 3-mo, n=6), 6-mo control (CTL 6-mo, n=12), 6-mo AAV1-shScramble (+ shSCR, n=11) and 6-mo AAV1-shAngptl2 (+ shAngptl2, n=12) ATX mice revealing white atheroma plaques at 6-mo. Data are mean±SEM. *: p<0.0001 *vs*. CTL 6-mo and shSCR; ^$^: p<0.0001 *vs.* CTL 3-mo.

Plaque was not present at 3-mo (Figures 1B and 4A), but the atherosclerotic lesion covered 8±1% of the thoracic aorta of 6-mo untreated ATX mice, and 8±1% of the thoracic aorta of mice injected with an AAV1 containing a scrambled (SCR; Table S1) mRNA sequence (Figure 4). At 6-mo, 3 months AAV1-shAngptl2 post-injection, the atherosclerotic burden in the thoracic aorta was reduced by 58% (Figure 4; p<0.0001; n=12) compared to un-injected ATX mice and mice injected with an AAV1-shSCR. Therefore, targeting *angptl2^+^* vascular cells is anti-atherogenic.

### Vascular *Angptl2* down-regulation decreases markers of senescence, monocytes recruitment and inflammation in the endothelium

**Figure 5:**
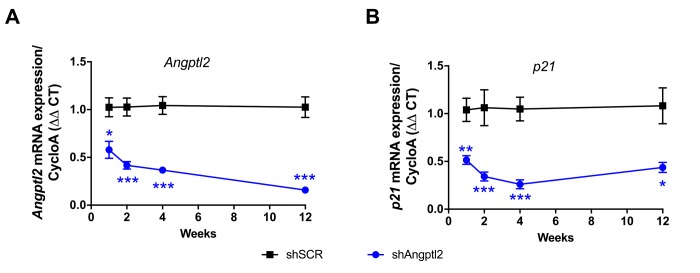
**Time-dependent decrease of senescent-associated *Angptl2* and *p21* mRNA expressions in** **the native endothelium of the aorta post-AAV1-shAngptl2 injection.** Decrease of *Angptl2* (**A**) and *p21* (**B**) mRNA expression post-injections (1-week, n=5-6; 2-week, n=5-7; 4-week, n=4-7; 3-month, n=6-4) in the native endothelium freshly harvested from thoracic aortas of ATX mice treated with AAV1-shSCR or AAV1-shAngptl2. Average gene expression level in shSCR group was arbitrarily set at 1. Data are mean±SEM. *: p<0.05; **: p<0.01; ***: p<0.001 *vs*. shSCR group, at each time point.

**Figure 6:**
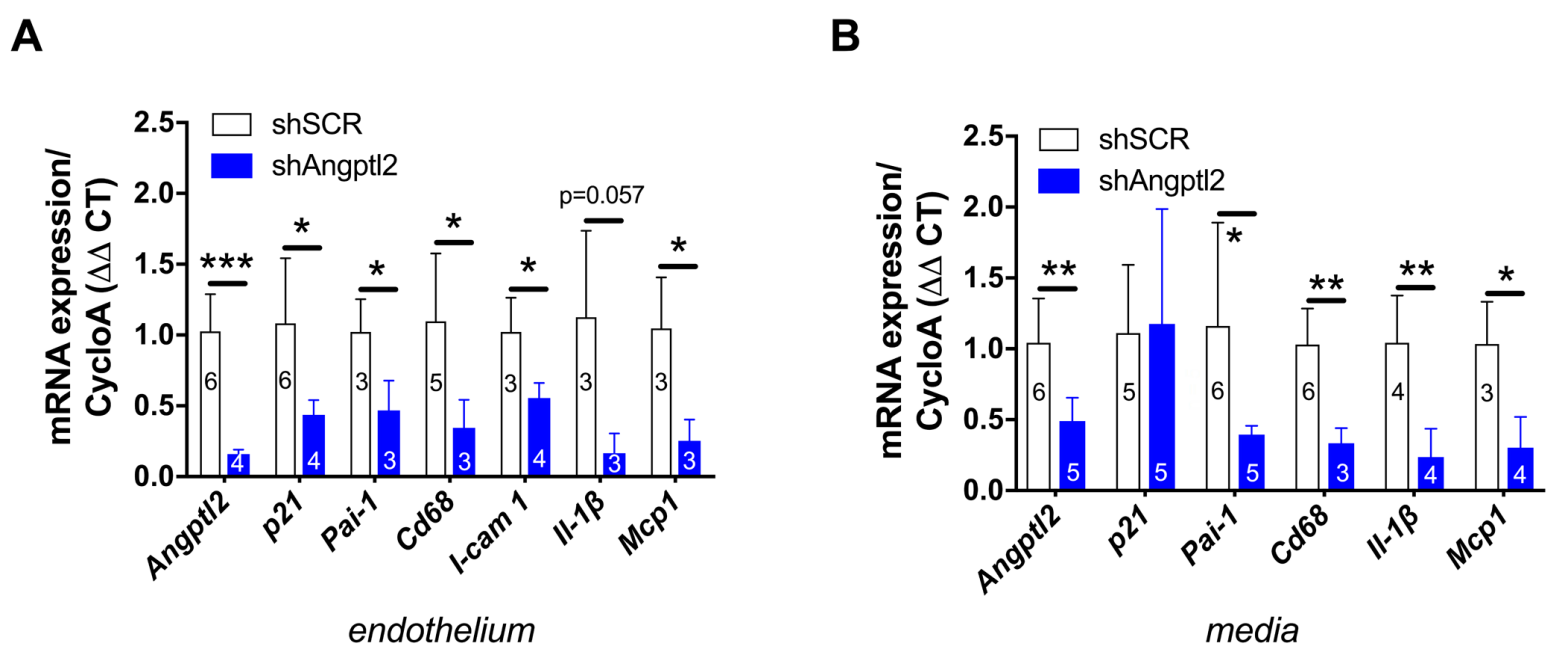
**Down-regulation of vascular *angptl2* is senolytic and anti-atherogenic.** (**A**) Decrease of mRNA expression of the indicated genes in the native aortic endothelium isolated from AAV1-shAngptl2-treated ATX mice, 3 months post-infection. Data are mean±SEM of n mice. *: p<0.05; **: p<0.01; ***: p<0.001 *vs*. shSCR group. (**B**) Decrease of mRNA expression (except for *p21*) in the de-endothelialized aortic wall (media) isolated from AAV1-shAngptl2-treated ATX mice, 3 months post-infection. Data are mean±SEM. *: p<0.05 and **p<0.01 *vs*. shSCR group.

Some ATX mice were sacrificed 1, 2 and 4 weeks after AAV1-shAngptl2 infection. After only one week, endothelial expression of *angptl2* and *p21* were significantly lowered and continued to decline over the 4-week period post-AAV1-shAngptl2 injection (Figure 5), demonstrating a fast reduction in senescence. At the end of the treatment, 3 months post-AAV1-shAngptl2 injection, *angptl2*, *p21* (Figures 5 and 6A) and *Pai-1* expressions were still significantly decreased in the endothelium (Figure 6A), suggesting that knockdown of *angptl2* lowers senescence in the long-term. Therefore, targeting *angptl2^+^* EC reduces senescent EC load *in vivo*; conversely, this suggests that *angptl2^+^* EC are senescent.

Senescent EC are known to favour adhesion of monocytes and their migration into the intima [37]. Accordingly, in the endothelium of 6-mo treated AAV1-shAngptl2 mice the expressions of the adhesion molecule *Icam-1* and of monocyte chemo-attractant protein *Mcp1* were reduced (Figure 6A) and this was associated with a lower expression of *Cd68^+^* in endothelial cells (Figure 6A); the expression of the inflammatory marker *IL-1β* also tented (p=0.057) to decrease in AAV1-shAngptl2 mice (Figure 6A). Altogether, these data suggest that targeting *angptl2^+^* senescent EC reduces inflammation and adhesion of *Cd68^+^* monocytes onto the endothelium.

### Vascular *Angptl2* down-regulation decreases inflammation, monocytes recruitment and macrophages infiltration into the media of the aorta

In the present study, *p21* expression was not altered in the media (including the atheroma plaque) of the aorta 3 months post-AAV1-shAngptl2 infection (Figure 6B), suggesting that in this mouse model and at this young age, arterial smooth muscle cells, fibroblasts and foam cells are not senescent. *Angptl2* and *Cd68* expression, however, decreased (Figure 6B), as did *Pai-1*, *Il-1β* and *Mcp1*, suggestive of less macrophage infiltration into the media and a lower inflammation. Indirectly, it also suggests that *Cd68^+^* macrophages express *angptl2* and contribute to the local inflammation, as previously reported [29,32,40]. Therefore, targeting *angptl2^+^* vascular cells reduces inflammation associated with macrophages infiltration into the media.

### Vascular *Angptl2* down-regulation induces early apoptosis of senescent ECs and promotes endothelium repair

**Figure 7:**
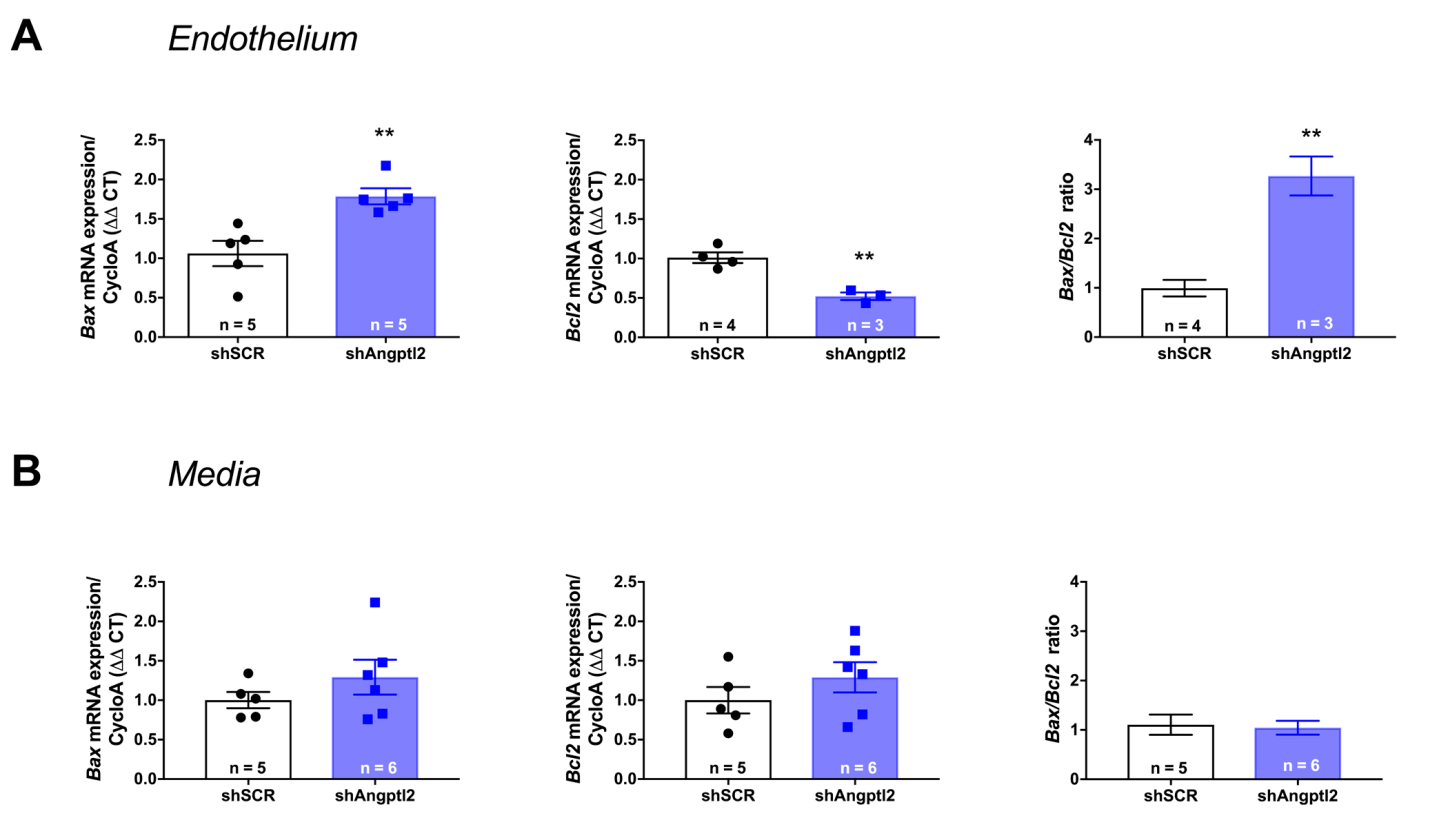
**Down-regulation of vascular *angptl2* is senolytic by promoting apopotosis.** (**A**) Induction of *Angptl2^+^* senescent endothelial cells apoptosis 1-week post-injection of AAV1-shAngptl2 in aortas of ATX mice: *Bax* mRNA expression increased (left, p=0.005) while *Bcl2* mRNA expression decreased (middle, p=0.003), increasing *Bax*/*Bcl2* ratio (right). (**B**) Absence of apoptosis in cells from the media 1-week post-injection of AAV1-shAngptl2 in aortas of ATX mice. Data are mean±SEM of n ATX mice.

To determine the mechanism of senescent EC clearance and endothelial repair associated with the anti-atherogenic effect of the shAngptl2, we euthanized ATX mice 1, 2 and 4 weeks after AAV1-shAngptl2 infection. Concomitantly with the fast reduction in senescence (Figure 5), after only one week post-infection, endothelial pro-apoptotic *Bax* expression was significantly increased (p=0.005), while that of the anti-apoptotic *Bcl2* was significantly decreased (p=0.003), increasing the *Bax*/*Bcl2* ratio (Figure 7A), suggesting that *angptl2^+^* senescent EC were eliminated, at least partly, following activation of this apoptotic pathway. In contrast, in the media neither *Bax* nor *Bcl2* expression changed one week post-infection (Figure 7B), and this absence of apoptosis paralleled the lack of reduction in the senescence marker *p21* in the media (Figure 6). Thus, AAV1-shAngptl2 infection promotes cell death of senescent *angptl2*^+^ endothelial cells by apoptosis; in contrast, in other cells from the aortic wall, shAngptl2 does not affect senescence and does not induce apoptosis.

**Figure 8:**
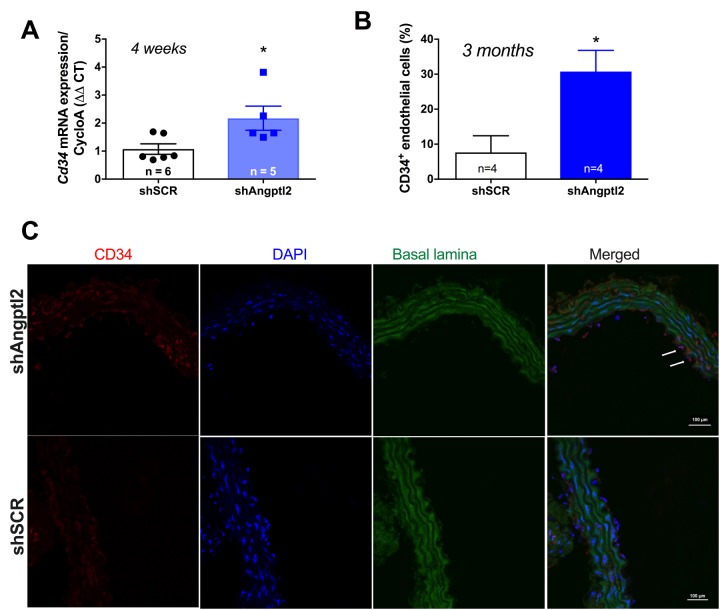
**Down-regulation of vascular *angptl2* is anti-atherogenic and promotes endothelial repair.** (**A**) Increased expression of the progenitor marker *Cd34* mRNA in the native aortic endothelium 4-week post-injection of shAngptl2 ATX mice. Data are mean±SEM. * : p<0.05 *vs*. shSCR group. The average gene expression levels in the shSCR group were arbitrarily set at 1. (**B**) Higher CD34 staining in the endothelium of 6-month old ATX mice 3 months post-injection of the AAV1-shAngptl2 compared to ATX mice injected with the scramble shRNA (shSCR). Data are mean±SEM. *: p<0.05 *vs*. shSCR. **C**) Representative images of CD34 immunostaining are shown.

Since the shAngptl2 therapy is anti-atherogenic, it would be expected that endothelial repair occurred as a mean to restore and/or maintain endothelial functions. After 2 weeks of shAngptl2 treatment, *Cd34* expression tended to increase in the endothelium (from 1.1±0.3 to 1.9±0.3, p=0.082, n=5 and 7, respectively; data not shown), and after 4 weeks *Cd34* expression increased significantly (p=0.034) (Figure 8A). This increase in the marker of progenitor cells, suggesting Cd34^+^ progenitor cell recruitment, was maintained up to the end of the study period, *i.e*. 3 months post-injection (Figure 8B-C). In contrast, endothelial apoptosis was no longer observed 3 months post-injection (data not shown). This strongly suggests that rapid apoptosis of *angptl2^+^* senescent EC stimulates long-term endothelial repair, at least in part by incorporating circulating endothelial progenitor cells (CD34^+^ EpC).

### Vascular *ANGPTL2* expression correlates with vascular senescence in internal mammary artery of patients with severe coronary artery disease

**Figure 9:**
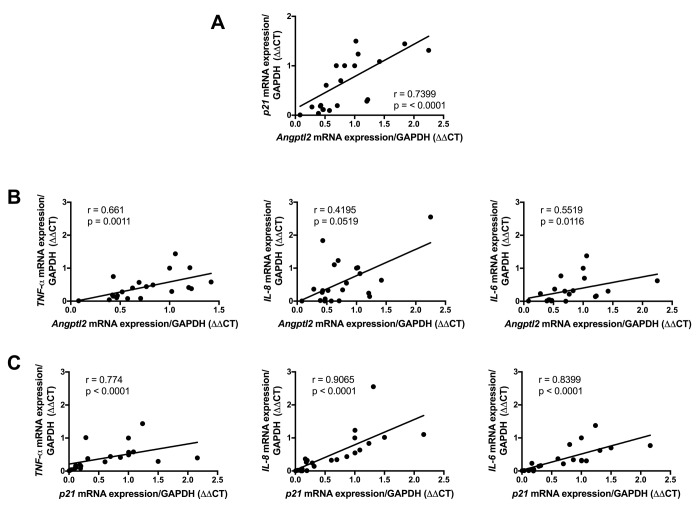
**Association of vascular ANGPTL2, senescence and inflammation in IMA from atherosclerotic patients undergoing coronary artery by-pass surgery.** Linear correlations between (**A**) *ANGPTL2* and *p21* mRNA expression, (**B**) *ANGPTL2* and *TNF-α,* *IL-8* and *IL-6* mRNA expression and (**C**) *p21* and *TNF-α,* *IL-8* and *IL-6* mRNA expression in IMA segments (n=26). Non-parametric Spearman correlation test was applied. Patient’s characteristics are described in Table S2.

To validate the clinical relevance of *ANGPTL2* as a marker of vascular senescence, and to assess whether accumulation of senescent cells precedes plaque growth in human arteries, we measured the expression of *ANGPTL2* and markers of senescence in human arterial segments. We recruited 26 patients undergoing coronary artery bypass (CABG) surgery with or without valve replacement (characteristics of the patients are in Table S2). In internal mammary artery segments free of plaque, the expression of *p21* and that of *ANGPTL2* were tightly associated (Figure 9A) and did not correlate with the age of the donor (data not shown). Furthermore, *ANGPTL2* and *p21* levels correlated with pro-inflammatory cytokines of the SASP, *i.e*., *TNFα*, *IL-8* and *IL-6* levels (Figure 9B-C). These clinical data validate the strong link between vascular *ANGPTL2* and the marker of senescence *p21,* in yet non-atherosclerotic arteries, independently of age. These data also suggest that accumulation of senescent vascular *ANGPTL2^+^;p21^+^* cells might precede plaque growth in human arteries.

## DISCUSSION

**Figure 10:**
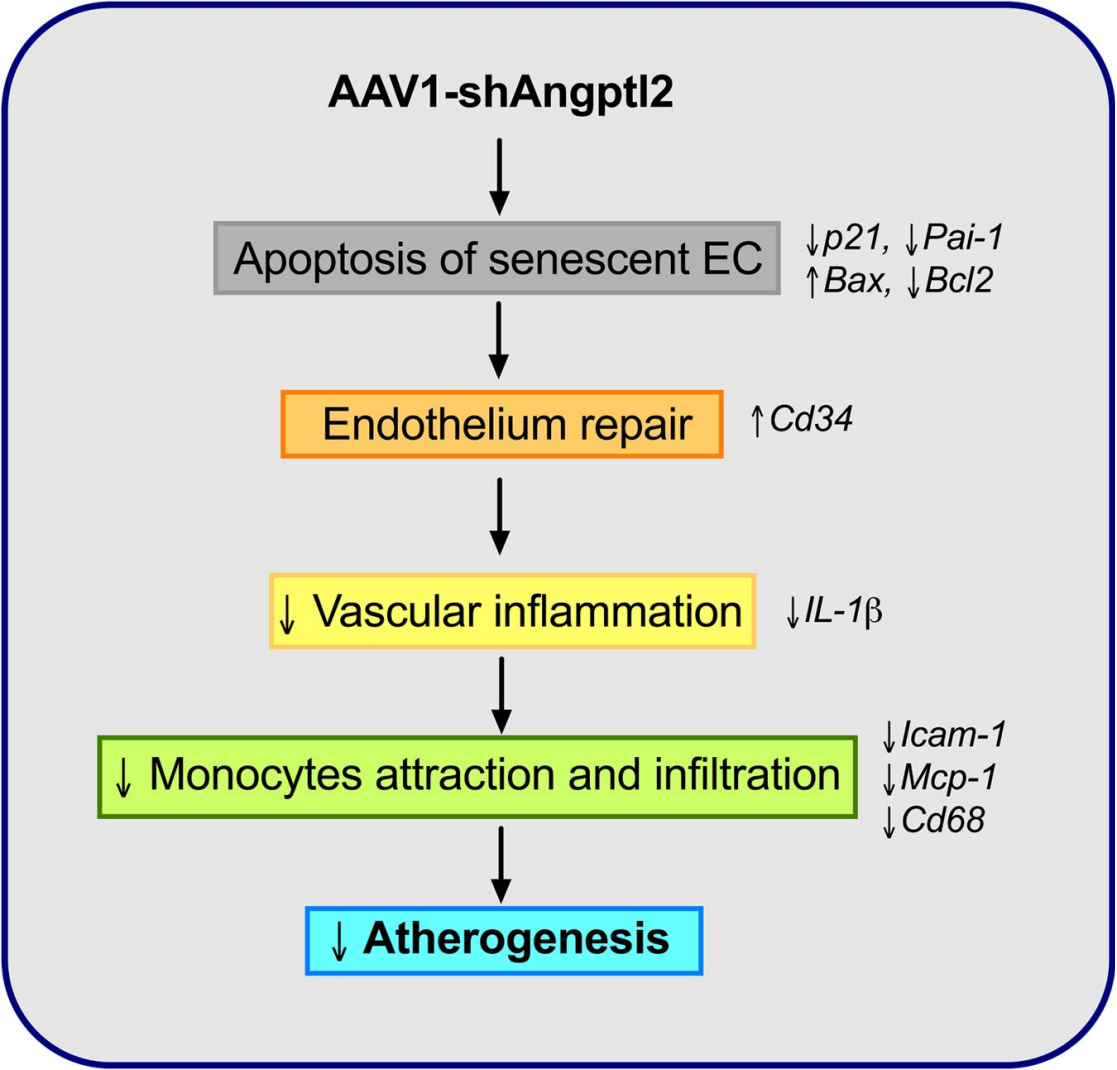
**Schematic representation of the putative anti-atherogenic action of AAV1-shAngptl2.** Elimination of *angptl2*^+^:*p21*^+^ senescent endothelial cells by apoptosis promotes endothelial repair. This leads to a reduced inflammatory profile, a lower immune cell adhesiveness and infiltration and therefore, a reduced atherogenicity.

The novel findings of this study are that down-regulation of vascular *Angptl2* may limit the progression of atherosclerotic lesions in pre-atherosclerotic dyslipidemic mice by 1) inducing clearance of senescent ECs through apoptosis and 2) by promoting endothelium repair and thus, attenuating the pro-inflammatory environment in the wall of the aorta. We have also shown that vascular *ANGPTL2* expression is correlated with senescence and inflammatory cytokines of the SASP in pre-atherosclerotic internal mammary artery from patients with coronary artery disease, suggesting that angptl2 is a clinical marker of arterial senescence and that arterial senescence may precede plaque growth in human arteries. Altogether, these observations demonstrate that targeting vascular *angptl2* is an innovative strategy to delay atherosclerosis by reducing endothelial senescence (Figure 10).

Age-related atherosclerosis is partly driven by the accumulation of senescent cells in mice [12,14], creating a pro-inflammatory environment through their SASP [1]. Targeting senescent cells using senolytic drugs appears therefore a promising strategy to reduce the burden of age-related chronic inflammatory diseases [15], including atherosclerosis [16,17]. Selective and safe senolytics have yet to be discovered as most come from the oncology therapeutic field [18]. The protein angptl2, on the other hand, is highly expressed by senescent vascular human EC [30], but not by quiescent or proliferative EC [31], and is highly atherogenic when infused in young LDLr^-/-^;hApoB100^-/-^ atherosclerotic mice [31]. Abundant ANGPTL2 expression was also observed in atheroma plaques of patients, particularly in EC and infiltrated macrophages [32]. The presence of senescent cells was also reported in human atherosclerotic arteries [17,41]. Although co-expression of vascular angptl2 and senescence markers has never been reported, our data show that by targeting *angptl2^+^* vascular cells, senescent cells are eliminated, and thus strongly suggest that angptl2 is a marker of arterial senescence. Indeed, our recent study demonstrated that the per-operative variations in circulating plasma levels of angptl2 following cardiac surgery were determined by fat and arterial tissue inflammatory and senescence load of the patients [36]. Therefore, angptl2 is a clear marker of aging, which levels of expression are magnified by the presence of risk factors for vascular diseases [42] and are linked to arterial senescence [36]. The demonstration that down-regulation of *angptl2* is anti-atherogenic therefore validates the concept of the contribution of senescent EC in the pathogenesis of atherosclerosis.

Several data collected in this work converge to support the contribution of *angptl2*^+^ senescent EC in endothelial dysfunction and in atherogenesis. Pro-inflammatory senescent EC are activated and promote aggregation of leukocytes [37], the initiating step of atherogenesis [38]. Our results reveal that following shAngptl2 treatment, the endothelial expression of the adhesion molecule *Icam-1* and the endothelial expression of monocyte chemo-attractant protein *Mcp1* were reduced, likely contributing to lower adhesion of *Cd68^+^* monocytes onto the endothelium. Monocytes adhesion is crucial for macrophage infiltration and atheroma growth [38]. Endothelial cells and macrophages are the primary cells that secrete angptl2 in the atherosclerotic lesion [32], and angptl2 activates macrophage recruitment [29]. Accordingly, reducing *angptl2^+^* senescent EC burden translates into lower EC activation, *i.e.* preserved endothelial anti-adhesive functions. Similarly, in hypercholesterolemic mice treated with the senolytic cocktail dasatinib/quercitin, endothelial dilatory function was improved *via* a greater nitric oxide (NO) bioavailability [12]. Interestingly, we reported that the dilatory eNOS-NO pathway was up-regulated in *angptl2* knockdown mice [43], and that in contrast, angptl2 induced endothelial dysfunction [44]. Senescence was not quantified in the latter studies [43,44], but altogether our data suggest that lowering *angptl2*, and thus senescence, protects the endothelial functions. In the present study, *p21* expression was not altered in the media of the aorta 3 months post-infection, suggesting that in this mouse model and at this young age, arterial smooth muscle cells, fibroblasts and foam cells are not senescent. In contrast, *Angptl2* and *Cd68* expression in the media decreased 3 months post-infection, as did *Pai-1*, *Il-1β* and *Mcp1*. On one hand, this result suggests that *Cd68^+^* macrophages express *angptl2* and contribute to the local inflammation, as previously reported [29,32,40] and, on the other hand, that elimination of senescent *angptl2^+^* EC reduced monocyte adhesion and macrophage infiltration. Macrophages appear unique as they may express *p16* as part of a physiological response to immune stimuli rather than through senescence [19,45,46]. Hence, reduced adhesion and infiltration of macrophages suggest improved endothelial functions 3 months post-AAV1-shAngptl2 therapy. Beyond the known benefits of eliminating senescent cells [18], the concomitant reduction both in the endothelium and the media of *Pai-1*, a well recognized SASP member and inducer of senescence [47], is clinically relevant: genetically driven high circulating levels of PAI-1 correlate with the severity of atherosclerosis [48], while conversely, a null mutation in its gene reduces markers of biological aging and increases longevity in humans [49].

A high level of senescent cells limits tissue remodelling and repair [50]. In contrast, parallel to the reduced endothelial expression of *angptl2* and *p21*, endothelial (but not in the media) pro-apoptotic *Bax* expression was significantly increased after one week of treatment, while that of the anti-apoptotic *Bcl2* was significantly decreased. These data suggest that *angptl2^+^*;*p21*^+^ senescent EC were eliminated following activation of this apoptotic pathway, which has been shown to be an effective mean of remodelling [51]. Selective induction of death by activation of apoptosis of senescent cells is the common mechanism of action of senolytics [12,14,19,52]. Thus, elimination of senescent *angptl2^+^* EC cells by apoptosis is a senolytic approach.

Since the shAngptl2 therapy is anti-atherogenic, preserves endothelial anti-adhesive functions and reduces arterial wall inflammation, endothelial repair should occur. One major mechanism of repair is the recruitment of CD34^+^ progenitor cells from the circulation, and more specifically endothelial progenitor cells for re-endothelialization in mice [53]. Conversely, a reduced number of circulating EpC has been associated with an increase in major advanced cardiac events in patients with CVD [54]. Two weeks after shAngptl2 treatment, *Cd34* expression tended to increase in the endothelium (data not shown), it increased significantly after 4 weeks and was sustained up to 3 months, suggesting that the shAngptl2 therapy stimulated repair of the endothelium. Thus, apoptosis of *angptl2^+^*;*p21*^+^ senescent EC likely stimulated endothelial repair, at least partly by incorporating circulating EpC. To our knowledge, this result is the first study to imply that integration of EpC into the native endothelium contributes to a functional endothelial repair post-senolytic therapy. Down-regulating vascular *angptl2* should be safe; angptl2-KO [29] and our Angptl2-KD mice are healthy and resist better to an inflammatory stress [44].

Senescent EC overlay the atheroma plaque in patients [17]. To validate the clinical relevance of *ANGPTL2* as a marker of vascular senescence, and to assess whether vascular senescence precedes plaque growth in patients, we measured the expression of *ANGPTL2* and markers of senescence in functional, not atherosclerotic internal mammary artery segments from patients with CVD. In these arterial segments, the expression of *p21* and that of *ANGPTL2* were tightly associated, independently of the age of the donor, demonstrating that accumulation of senescent vascular cells is driven by stress (risk factors for CVD) more than age *per se*, as we previously reported in cultured EC isolated from mammary arteries [55]. The strong levels of association of *ANGPTL2* and *p21* levels with the pro-inflammatory cytokines of the SASP, *i.e*., *TNFα*, *IL-8* and *IL-6* levels strengthen the concept that senescence-driven chronic low-grade inflammation ultimately triggers the appearance of the atheroma with time.

Limitations of the study: first, almost all data were provided on mRNA level. Unfortunately, there was not enough material in the native endothelium layer scraped from the mouse aorta to look at the protein level. Second, *n* number is relatively small and variable due to the limited and variable availability of endothelial mRNA extractions. Third, expression of *p21* mRNA was unaffected by the shAngptl2 in the media, suggesting either that cells in the media were not senescent, as we propose in these young ATX mice, or, alternatively, that angptl2 is not central to the maintenance of senescence in cells other than endothelial cells. Finally, the underlying mechanism of elevated CD34^+^ progenitor cell recruitment after shAngptl2 treatment was not elucidated. Because on one hand, angptl2 is anti-apoptotic [56], and because on the other hand, the shAngptl2 induced apoptosis of endothelial cells, it is possible that the generation of apoptotic bodies act as chemoattractant for EpC [57,58]. This attractive hypothesis would need to be validated in specific experiments.

In conclusion, therapeutic down-regulation of vascular *angptl2* leads to the clearance of senescent EC by apoptosis, stimulates endothelial repair, preserves endothelial functions and reduces atherosclerosis. *ANGPTL2* and *p21* expression are tightly associated in non-atherosclerotic human arteries, which further suggests that accumulation of arterial senescence precedes age-related atherosclerosis. Altogether, these data suggest that targeting vascular *angptl2* could be a selective and safe senolytic strategy to delay or reduce atherogenesis.

## MATERIALS AND METHODS

### Animal study

All animal experiments were performed in accordance with the “Guide for the Care and Use of Experimental Animals of the Canadian Council on Animal Care” and the “Guide for the Care and Use of Laboratory Animals” of the US National Institutes of Health (NIH Publication No. 85-23, revised 1996) and was approved by the Montreal Heart Institute Ethics Committee. Knockout/transgenic severely dyslipidemic LDLr^-/-^;hApoB^+/+^ (ATX) male mice were fed a normal diet. Mice were anaesthetized with 44 mg/kg ketamine and 2.2 mg/kg xylazine and ventilated. Thoracic aortas of ATX and WT mice were used to quantify atherosclerotic lesions and to assess mRNA expression of senescent markers, inflammatory markers, progenitor marker and apoptosis markers both in the freshly isolated native endothelium and in the aorta wall [31]. Hearts, livers and blood of ATX mice were used to validate the vascular tropism of the AAV1.

### AAV1 production

The protocol was adapted from a previous study [39]. HEKT293T competent cells were plated until a confluence of 70%, and were then transfected overnight with 12 µg/mL of the pXYZ C1 plasmid vector (serotype 1), 4 µg/mL of the plasmid containing shAngptl2, shSCR (see shRNA sequences Table S1) or mCherry red fluorescent protein sequence, and 48 µg/mL of polyethylenimin (Sigma) in a starvation medium. The next day, the medium was changed and cells were incubated for 48h with normal growth medium. Then, cells were collected and lysed with Tris 1M/ NaCl 5M pH 8.5. Lysis was accelerated by several freeze/thaw steps and 1 µL of MgCl_2_ (1M) in addition to benzonase (250U/µL, Sigma) were added *per* mL of lysate. After centrifugation, AAV1 were isolated from the supernatant by iodixanol gradients during ultracentrifugation, then extracted with a syringe and concentrated in a PBST buffer volume between 0.25 ml and 0.5 ml with Ultracel-100 regenerated cellulose membrane (100 kDa, EMD Millipore).

### AAV1 titration and administration in ATX mice

Titration was performed by qPCR reactions using a StepOnePlus Real-Time PCR System (Thermo Fisher Scientific). AAV1 were quantified using a standard range made by serial dilutions (10 ng to 10^-6^ ng) of shAngptl2 and shSCR plasmids containing a target sequence (BGH) or the plasmid containing mCherry sequence. The primers for BGH target sequence and *mCherry* were designed using Clone Manager software (Table S3). Each mouse received a systemic (i.v.) bolus injection of 10^11^ AAV1 particles at 3-month old (-mo) and were studied at 6-mo; some mice received an injection at 5-mo and were studied after 1 week, 2 weeks or 4 weeks. A pilot study showed that injection of 5x10^10^ AAV1 particles was sub-optimal, leading to an inconsistent reduction in *angptl2* by ∼50% (data not shown).

### Quantification of atherosclerotic lesions

Freshly isolated thoracic aortas of ATX mice were longitudinally opened and pictures taken with a digital camera. Atherosclerosis lesions were quantified by measuring the white spots in the aorta using ImageJ software as previously [31]. Plaque areas were expressed as percentage of total aortic area.

### Total RNA extraction

Total RNA was extracted from freshly isolated aorta, separately in both the endothelial layer and the arterial wall. Native ECs were gently scraped with the tip of a scalpel blade from longitudinally opened segments of the thoracic aorta and endothelial mRNA was extracted as previously [31]; the denuded aorta wall (including the atheroma plaque), the liver and the heart were pulverized in liquid nitrogen with a Cell crusher kit (Cellcrusher Limited). RNAs were extracted using an RNeasy Mini Kit (Qiagen). Contamination with genomic DNA was prevented by a digestion with a DNase I mix (Qiagen), according to the manufacturer’s guidelines. Total RNA was quantified using a NanoDrop ND-100 spectrophotometer.

### Real-Time quantitative PCR

Total RNA was reverse transcribed into first-strand complementary DNA with M-MLV reverse transcriptase (Thermo Fisher), using random hexamer primers. The qPCR reactions were carried out on diluted RT products by using the DNA-binding dye SYBR Green PCR Master Mix (Thermo Fisher Scientific) to detect PCR products with StepOnePlus Real-Time PCR System (Thermo Fisher Scientific). The primers of target genes (*Angptl2, p21, Il-1β, Icam-1, Cd68, Cd34, Mcp1, Bax, Bcl2*) were designed using Clone Manager software (Table S3 (mouse) and S4 (human)). All samples were run in duplicate and the fold changes in gene expression were calculated by a ΔΔC_T_ method using *cycloA* (cyclophilin A) as the housekeeping gene. N numbers are not homogeneous due to the limited and variable availability of endothelial mRNA extraction.

### Immunofluorescence

Fixed tissues (transversely aortic frozen sections) were incubated with 1:100 diluted rabbit anti-mCherry (#ab167453; Abcam) or with 1:200 diluted rabbit anti-CD34 (#ab185732; Abcam) and 1:800 diluted secondary antibody Alexa fluor-647 anti-rabbit (#A31573, Thermo Fisher Scientific). DNA counterstaining was performed by incubating fixed tissues with DAPI (D1306, Thermo Fisher Scientific). Fluorescence was visualized by confocal microscopy (Zeiss LSM 510) with 40X magnification for mCherry staining and 20X magnification for CD34 staining. CD34 staining was normalized to the total number of nuclei present in the endothelial layer (DAPI staining).

### Biochemical analysis

Blood was rapidly collected after sacrifices and then centrifuged within minutes to collect the plasma. Samples were stored frozen in aliquots at -80°C until further analysis. Plasma metabolic profile (total cholesterol, glucose and triglycerides) was measured at the biochemical laboratory of the Montreal Heart Institute.

### Human study

Twenty-six coronary artery disease (CAD) patients undergoing coronary arterial bypass (CABG, n=16), or both CABG and aortic or mitral valve replacement (VR) (n=10) between February 2014 and January 2016, were prospectively included in the study at the Montreal Heart Institute (Quebec, Canada). Inclusion criteria were patients undergoing elective CABG and VR with normal left ventricular function at pre-operative evaluation. Patients who underwent other type of procedure were excluded. All patients signed an informed consent and the study (#ICM 13-1492) was approved by the ethical committee on human research of the Montreal Heart Institute. On the overall population, the mean age was 67±8 years and 92% were male. All patients had at least one risk factor for CVD and received preventive treatment such aspirin or statin before surgery. Left ventricular function was normal in all patients. The basal pre-operative characteristics of the patients are detailed in the Table S2. All patients underwent cardiac surgery with cardiopulmonary bypass (CPB). During surgery, a distal segment of left internal mammary artery (IMA) measuring at least 1 cm in length was harvested and stored at -80°C for all patients. Then gene expression of *ANGPTL2* and other markers of inflammation (*TNFα*, *IL-8*, *IL-6*) and senescence (*p21*) were measured in this tissue. Frozen IMA fragments were pulverized in liquid nitrogen and dry ice with a Cell crusher kit (Cellcrusher Limited). Samples of organ powder were then processed for RNA extraction according to the manufacturer's instructions (RNeasy mini kit. Qiagen). Contamination with DNA was prevented by a digestion with a DNase I mix (Qiagen) according to the manufacturer’s guidelines. Total RNA was quantified using a NanoDrop ND-100 spectrophotometer. Total RNA was reverse-transcribed into first-strand complementary DNA with M-MLV reverse transcriptase (Thermo Fisher Scientific) using random hexamer primers. The primers of target genes were designed using Clone Manager software (Table S4). Quantitative polymerase chain reaction was performed using the SYBR Green PCR Master Mix (Thermo Fisher Scientific). All samples were run in duplicate and the fold changes in gene expression were calculated by a ΔΔCT method using *GAPDH* as the housekeeping gene.

### Statistical analysis

Data are presented as mean±SEM, with “*n*” indicating the number of animals or patients. Animal data were analyzed using parametric and non-parametric tests: a two-tailed unpaired *t* test was used to compare plaque growth and endothelial gene (*Angptl2,* *p21* and *Pai-1*) expression in ATX mice at 3- *vs*. 12-mo (Figure 1). A two-way ANOVA with Sidak’s multiple comparisons test was performed to compare mRNA expressions in WT and ATX mice (Figure 2). One-way ANOVA with Tukey’s posthoc-test was used to compare plaque area in untreated 3-mo, AAV1-treated or -untreated 6-mo ATX mice (Figure 4). Unpaired *t* test or Mann Whitney test was used to compare mRNA expression in shSCR group (control) *versus* shAngptl2 group (Figures 5, 6, 7 and 8). Human data are presented as dot plots. Non-parametric Spearman correlation analyses were performed to correlate the mRNA levels of *ANGPTL2* and *p21* with *TNFα*, *IL-8* and *IL-6* in IMA segments from patients (Figure 9). All statistics were performed using Graph Pad Prism 7.0.

## Supplementary Material

Supplementary Tables
