# Supplementary material for: Knockdown of angiopoietin-like 2 induces clearance of vascular endothelial senescent cells by apoptosis, promotes endothelial repair and slows atherogenesis in mice
Source: Aging (Albany NY). 2019 Jun 11;11(11):3832–50. doi: 10.18632/aging.102020 (PMC6594793; doi:10.18632/aging.102020)
Supplement: Supplementary Tables [file aging-11-102020-s001.pdf]

SUPPLEMENTARY TABLES

Table S1. Sequences of the shRNAs.

shRNA	sequence
Angptl2	GCAGAGTCTTCCAATCAGTTAATCAAGAGTTAACTGATTGGAAGACTCTGC
Scramble*	CCTAAGGTTAAGTCGCCCTCGCTCGAGCGAGGGCGACTTAACCTTAGG

* The scramble sequence is directed against *luciferase* not expressed in mammals.

Table S2. Clinical profiles of the atherosclerotic patients undergoing artery bypass graft surgery and/or valve replacement.

	All CAD patients	Valve +CABG	CABG
	n=26	*n*=10	*n*=16
Age (years)	67±2	69±2	65±2
Sex (men, %)	24 (92%)	10 (100%)	14 (88%)
Height (m)	1.71±0.02	1.68±0.01	1.73±0.03
Weight (kg)	84.6±2.5	84.1±3.8	85.0±3.5
BMI (kg/m^2)	28.9±0.8	29.8±2.0	28.3±0.9
**Risk factors (*n*, %)**			
Dyslipidemia	24 (92%)	8 (80%)	16 (100%)
Hypertension	22 (85%)	9 (90%)	13 (81%)
Obesity	12 (46%)	6 (60%)	6 (38%)
Diabetes	11 (42%)	3 (30%)	8 (50%)
Active smoker	3 (12%)	1 (10%)	2 (12%)
Ex-smoker	9 (35%)	4 (40%)	5 (31%)
COPD	2 (8%)	2 (20%)	0 (0%)
Family history of CVD	9 (35%)	4 (40%)	5 (31%)
**Medication**			
β-blockers	22 (85%)	8 (80%)	14 (88%)
ACE inhibitors	4 (15%)	1 (10%)	3 (29%)
AR antagonists	6 (23%)	3 (30%)	3 (29%)

Diuretic	9 (35%)	3 (30%)	6 (38%)
Hypoglycemic agent	9 (35%)	1 (10%)	8 (50%)
Anticoagulant	4 (15%)	1 (10%)	3 (19%)
Aspirin	24 (92%)	8 (80%)	16 (100%)
Statin	25 (96%)	9 (90%)	16 (100%)
**Blood analysis**			
Total cholesterol (mmol/L)	3.3±0.2	3.3±0.3	3.2±0.3
Cholesterol-LDL (mmol/L)	1.5±0.2	1.5±0.2	1.5±0.3
Cholesterol-HDL (mmol/L)	1.2±0.07	1.0±0.1	1.3±0.09
Triglycerides (mmol/L)	1.4±0.1	1.7±0.3	1.2±0.1
HbA1C (%)	0.064±0.008 (11)	0.059±0.03 (3)	0.066±0.003 (8)

Data are mean±SEM of (*n*) patients. No significant difference between groups (One-way ANOVA followed by Tukey's post hoc test, or Kruskal Wallis followed by Dunn's post hoc test for non Gaussian distribution).

Abbreviations: ACE inhibitors, angiotensin converting enzyme inhibitors; AR antagonists, angiotensin II receptors antagonists; BMI, body mass index; CAD, coronary artery disease; COPD, chronic obstructive pulmonary disease; CVD, cardiovascular disease; HbA1C, glycated hemoglobin A1C.

Table S3. Mouse primers used to quantify gene expression using quantitative RT-PCR.

Target gene	Forward	Reverse
Angptl2	GATCCAGAGTGACCAGAATC	TCTCAGGCTTCACCAGGTAG
Bax	AGCAAACTGGTGCTCAAGGC	CCACAAAGATGGTCACTGTC
Bcl2	GTGGTGGAGGAACTCTTCAG	GTTCCACAAAGGCATCCCAG
BGH	TGCCTTCCTTGACCCT	CCTTGCTGTCCTGCCC
Cd34	TGAGATGACATCACCCACCG	GCCAACCTCACTTCTCGGAT
Cd68	CATCAGAGCCCGAGTACAGTCTACC	AATTCTGCGCCATGAATGTCC
CycloA	CCGATGACGAGCCCTTGG	GCCGCCAGTGCCATTATG
Icam-1	CAATTCACACTGAATGCCAGCTC	CAAGCAGTCCGTCTCGTCCA
Il-1β	TGCCACCTTTTGACAGTGATG	TGATGTGCTGCTGCGAGATT
Mcherry	AGGTCAAGACCACCTAAAA	CTGTTCCACGATGGTGTAGT
Mcp1	GCAGGTCCCTGTCATGCTTC	CTCTCCAGCCTACTCATTGGG
p21	TGTCGCTGTCTTGCACTCT	AGACCAATCTGCGCTTGGA
Pai-1	TTGTCCAGCGGGACCTAGAG	AAGTCCACCTGTTTCACCATAGTCT

Angptl2, angiopoietin-like 2; Icam-1, intracellular adhesion molecule; IL-1β, interleukin 1β; Mcp1, Monocyte chemoattractant protein 1; CycloA, cyclophilin A.

Table S4. Human primers used to quantify gene expression using quantitative RT-PCR.

Target gene	Forward	Reverse
ANGPTL2	CCCCAACACCTTCCACTAAG	AACAGAATCCAGCATCCCG
TNFα	CTCTTCTGCCTGCTGCACTT	CTCTCAGCTCCACGCCATTG
IL-8	CTCTTGGCAGCCTTCCTGAT	TTCTGTGTTGGCGCAGTGTG
IL-6	GACAGCCACTCACCTCTTCA	CACCAGGCAAGTCTCCTCAT
p21	GGACCTGTCACTGTCTTGTA	CCTCTTGGAGAAGATCAGCCG
GAPDH	AATCCCATCACCATCTTCCA	AAATGAGCCCCAGCCTTC

ANGPTL2, angiopoietin-like 2; TNF-α, Tumor necrosis factor; IL-8, interleukin 8; IL-6, interleukin 6.
